# *Arx* Expression Suppresses Ventralization of the Developing Dorsal Forebrain

**DOI:** 10.1038/s41598-018-36194-6

**Published:** 2019-01-18

**Authors:** Youngshin Lim, Il-Taeg Cho, Xiuyu Shi, Judith B. Grinspan, Ginam Cho, Jeffrey A. Golden

**Affiliations:** 1 Department of Pathology, Brigham and Women’s Hospital, Harvard Medical School, Boston, MA 02115 USA; 2 School of Life Sciences, Xiamen University, Xiamen, Fujian, 361005 China; 3 Department of Neurology, Children’s Hospital of Philadelphia, University of Pennsylvania Perelman School of Medicine, Philadelphia, PA 19104 USA

## Abstract

Early brain development requires a tight orchestration between neural tube patterning and growth. How pattern formation and brain growth are coordinated is incompletely understood. Previously we showed that aristaless-related homeobox (ARX), a paired-like transcription factor, regulates cortical progenitor pool expansion by repressing an inhibitor of cell cycle progression. Here we show that ARX participates in establishing dorsoventral identity in the mouse forebrain. In *Arx* mutant mice, ventral genes, including *Olig2*, are ectopically expressed dorsally. Furthermore, *Gli1* is upregulated, suggesting an ectopic activation of SHH signaling. We show that the ectopic *Olig2* expression can be repressed by blocking SHH signaling, implicating a role for SHH signaling in *Olig2* induction. We further demonstrate that the ectopic *Olig2* accounts for the reduced *Pax6* and *Tbr2* expression, both dorsal specific genes essential for cortical progenitor cell proliferation. These data suggest a link between the control of dorsoventral identity of progenitor cells and the control of their proliferation. In summary, our data demonstrate that ARX functions in a gene regulatory network integrating normal forebrain patterning and growth, providing important insight into how mutations in *ARX* can disrupt multiple aspects of brain development and thus generate a wide spectrum of neurodevelopmental phenotypes observed in human patients.

## Introduction

The establishment of dorsoventral (DV) identity in the developing neural tube enables the formation of separable progenitor zones and ultimately the generation of distinct neural subtypes. For example, dorsal forebrain progenitors produce excitatory (glutamatergic) projection neurons that make up approximately 80% of the neurons in the mature cerebral cortex^1,2^. In contrast, inhibitory interneurons, which use γ-aminobutyric acid (GABA) as a neurotransmitter, originate from the ganglionic eminences (GE) in the ventral forebrain and migrate dorsally to the cerebral cortex, making up approximately 20% of cortical neurons^3,4^. In addition to establishing the DV axis, the neural tube also undergoes substantial expansion of progenitor populations, a function that ultimately contributes to forebrain size^5^. Interestingly, multiple genes involved in early DV patterning also play important roles in the control of brain size^6,7^.

ARX is a vertebrate homologue of *Drosophila* aristaless (Al), a paired-like homeodomain transcription factor (TF). Mutations in *al* result in pattern disruptions in a subset of appendages of the adult fly^8^. The affected appendages show reduced size, which led to the speculation that *al* may also be a ‘region specific growth control gene’^8^. In fact, it has been shown that *al* is required for the growth and differentiation of the tip of the developing leg^9^. In developing mice, ARX is expressed in the progenitor cells located both in the ventricular zone (VZ) of the embryonic cortex (dorsal forebrain) and in the subventricular zone (SVZ) of the GE (ventral forebrain)^10,11^. In the GE, its expression is maintained even after the cells undergo migration and differentiation, while its dorsal expression is restricted to progenitor cells^12^. Patients with mutations in *ARX* present with intellectual disability and epilepsy, with or without structural defects in the brain such as lissencephaly (smooth brain), microcephaly (small brain), and agenesis of the corpus callosum, as well as abnormal genitalia^13–15^. These human phenotypes have largely been recapitulated in genetic mouse models, supporting a direct role of *ARX/Arx* mutations in the pathogenesis of this wide spectrum of phenotypes^15,16^.

Using a dorsal forebrain specific *Arx* mutant male mice (*Arx*^*flox/y*^*; Emx1*^*cre*^) (*ARX*/*Arx* is on the X-chromosome), we have previously shown that ARX modulates cortical progenitor proliferation and neurogenesis by directly repressing the expression of *Cdkn1c* (*Kip2*), a cell-cycle inhibitor gene^17^. Progenitor cells deficient for *Arx* prematurely exit the cell cycle, resulting in depletion of the proliferating progenitor cell pool and a reduction in upper layer neurons^17^. This has been postulated as the mechanism for the reduced brain size (microcephaly) reported in mice as well as in patients^14–20^.

In the present study, we show that the loss of *Arx* from the dorsal forebrain results in DV gene expression defects. A subset of predominantly ventral genes, including *Olig2*, are aberrantly overexpressed in the dorsal forebrain. OLIG2 is known as a ‘multifaceted TF’ that promotes neuronal and oligodendrocyte fates, and directs both differentiation and proliferation based on spatial and temporal dependent expression^21–24^. Our data reveal that the aberrant induction of *Olig2* leads to a reduction in PAX6 and TBR2, both dorsally restricted TFs crucial for proliferation and/or differentiation of the cortical progenitor cells. Our findings further indicate that ARX can regulate the specification of cortical progenitors by suppressing ventral identity while promoting dorsal identity. Taken together, we propose that ARX coordinates telencephalic patterning and forebrain size by regulating DV gene expression, including the suppression of dorsal *Olig2*, which modulates the expression of genes including *Pax6* and *Tbr2*, ultimately influencing forebrain patterning and growth.

## Results

### *Olig2* is ectopically expressed in ARX-deficient dorsal forebrain progenitors

**Figure 1:**
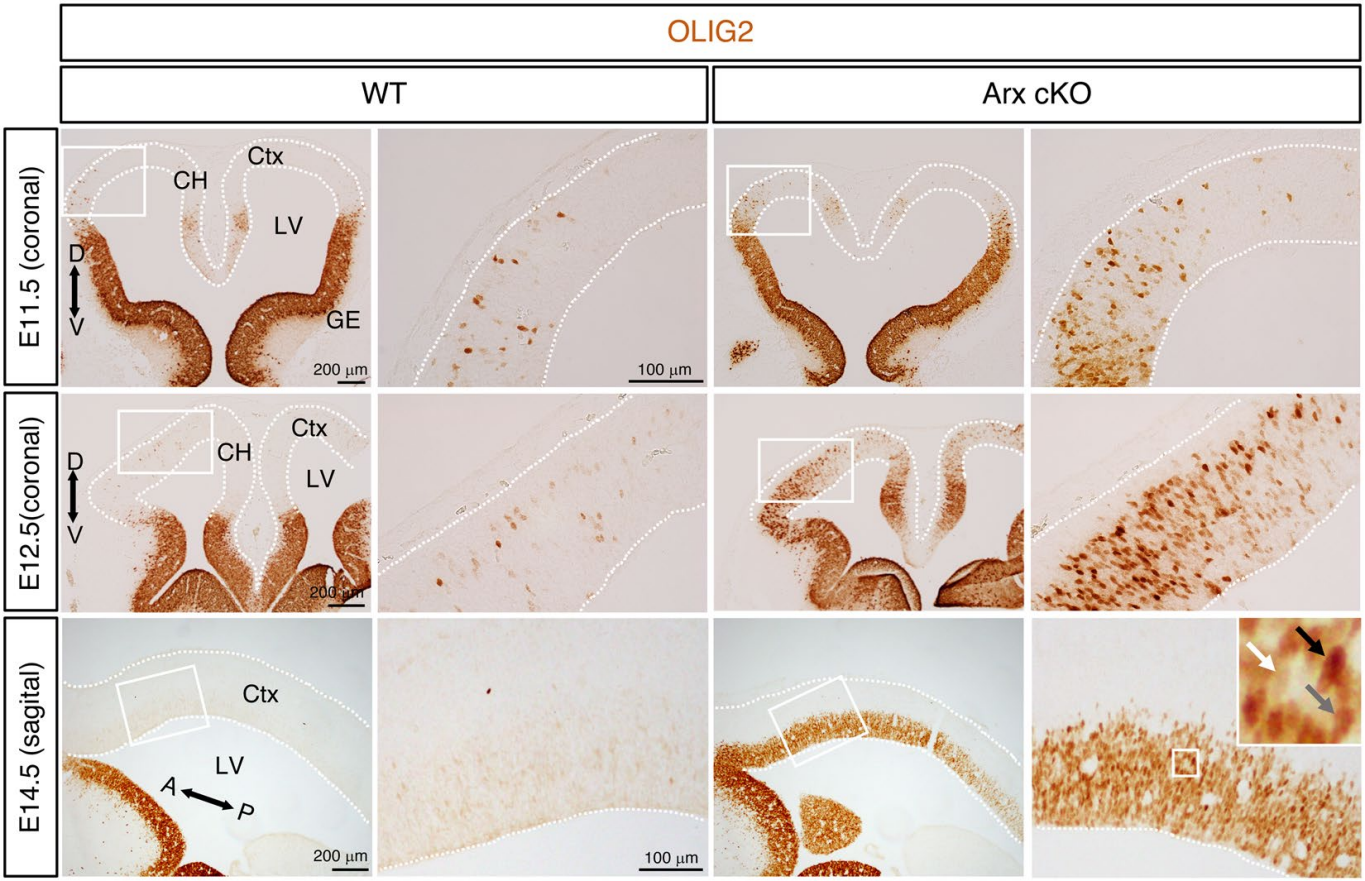
*Olig2* is ectopically expressed in *Arx*-deficient cortical epithelium. Representative images of embryonic neocortex of the *Arx*^+/*y*^ (WT) and *Arx*^*cKO/y*^ (*Arx* cKO) mice at E11.5, E12.5 (coronal sections) and E14.5 (sagittal sections) immunolabeled with OLIG2 antibody. White boxed areas in the left panels are displayed as magnified images in the right panels. Dotted lines mark the boundaries of the neural tube. A-P and D-V indicate anterior-posterior and dorsal-ventral axes, respectively. Three arrows in the inset (magnified image of the boxed area) of the right-bottom panel indicate cells with variable levels of OLIG2 expression (white, low; gray, intermediate; black, high). CH, cortical hem; Ctx, neocortex; GE, ganglionic eminence.

We previously identified 83 differentially expressed genes in the *Arx*^−/*y*^ cerebral cortex by microarray analysis (embryonic day 14.5, E14.5) and validated a subset by reverse transcription-quantitative real time PCR (RT-qPCR)^17^. Among the validated genes *Olig2* showed the highest upregulation^17^. To confirm this finding, we compared OLIG2 immunostaining from wild type (WT) (*Arx*^+/*y*^*; Emx1*^*cre*^, also referred to as *Arx*^+/*y*^) and *Arx* cKO (*Arx*^*flox/y*^*; Emx1*^*cre*^, also referred to as *Arx*^*cKO/y*^) embryonic brain sections. In WT mice, OLIG2 expression was strongly detected in the ventral forebrain (GE) at E11.5-E14.5, as expected (Fig. 1), although weak expression in a relatively small subset of progenitor cells was also seen dorsally (see WT boxed areas in Fig. 1), predominately in the anterior forebrain. In *Arx* cKO mice, OLIG2 staining in the ventral forebrain (GE) was similar to that observed in WT mice (Fig. 1). In contrast, OLIG2 expression in the dorsal forebrain (see cKO boxed areas in Fig. 1) was markedly increased, when compared to the WT brains, at E11.5-16.5 (Fig. 1 and Supplementary Fig. S1a). Given that CRE-mediated recombination only occurs in the dorsal but not in the ventral forebrain, at the *Arx* locus in *Arx*^*flox/y*^*; Emx1*^*cre*^ mice, our results indicate that the loss of *Arx* in the dorsal forebrain leads to a dramatic increase in OLIG2 positive cells in the dorsal forebrain. Interestingly, this abnormal OLIG2 expression exhibits a strong anterior-high to posterior-low gradient (Fig. 1), and variable levels in different cells (compare three arrows in Fig. 1 inset).

We next sought to establish if the OLIG2 positive cells result from ectopic expression of *Olig2*, in the dorsal forebrain, or from increased migration of ventrally derived OLIG2 positive cells. To distinguish these possibilities, we electroporated a GFP expression construct *in utero* to the cortical ventricular zone (VZ) of the *Arx*^*cKO/y*^ brains to mark dorsally positioned progenitor cells. We found that OLIG2 positive cells were also labeled with GFP, ensuring that these cells originated from the dorsal forebrain and not from the ventral GE (Supplementary Fig. S1b). Together these data indicate that the cortical progenitor cells in the dorsal forebrain of the *Arx* cKO mice abnormally overexpress OLIG2, which is normally repressed by ARX.

### ARX represses *Olig2* expression

**Figure 2:**
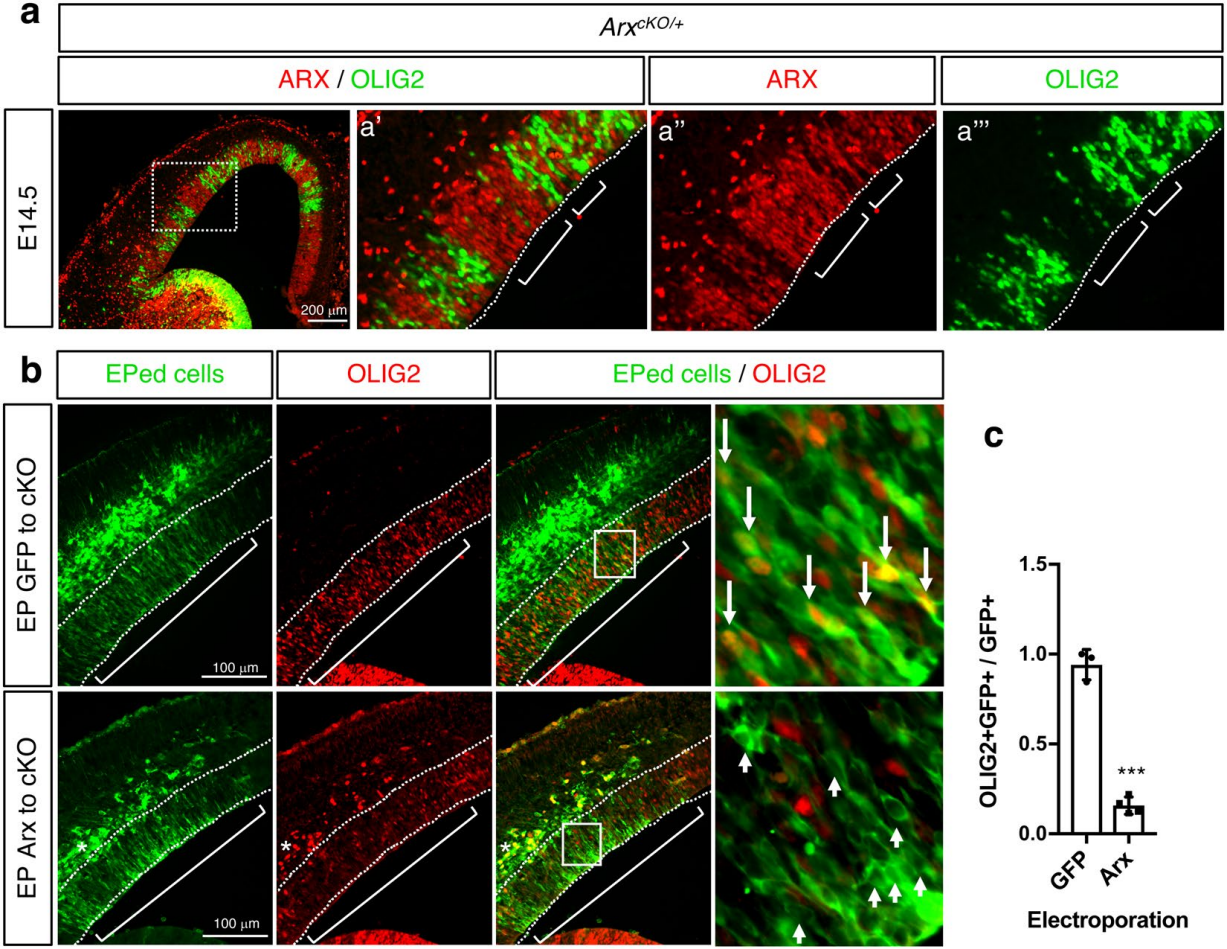
ARX represses OLIG2 expression. (**a**) Representative images of embryonic neocortex (E14.5) of the *Arx*^*cKO*/+^ female (Arx Het) double immunolabeled with OLIG2 and ARX antibodies (**a’**–**a”’**: magnified images of the boxed area in left panel). ARX+ cells are OLIG2−, and OLIG2+ cells are Arx−. Dotted lines mark the ventricular surface. Longer brackets indicate example areas with high number of ARX+ cells, while shorter brackets denote areas with high number of OLIG2+ cells. (**b**) Representative images of the immunofluorescent labeling (GFP and OLIG2) of the *Arx*^*cKO/y*^ cortex electroporated with either *pCIG* (encoding GFP only) or *pCIG-Arx* (encoding ARX-IRES-GFP) (EP at E13.5 and harvested at E14.5). GFP antibody was used to label electroporated (EPed) cells (green, cytoplasmic staining pattern). Right most images are magnified images of the boxed areas. Long arrows indicate examples of GFP electroporated cells expressing OLIG2 and short arrows indicate examples of ARX electroporated cells not expressing OLIG2. (**c**) Quantification of results in B. The ratio of OLIG2+ GFP+ cells over GFP+ cells was plotted for both GFP (*pCIG*) and Arx (*pCIP-Arx*) electroporated samples. Error bars: mean ± s.d (n = 3 for GFP EP and n = 4 for Arx EP; ***P = 0.0008; unpaired t-test).

To determine if the abnormal OLIG2 expression is a result of cell autonomous or non-autonomous function of ARX, we used *Arx* Het female (*Arx*^*flox*/+^; *Emx1*^*cre*^, also referred to as *Arx*^*cKO*/+^) brains where ARX positive cell columns are clonally distributed adjacent to ARX negative cell columns in the VZ of the dorsal forebrain due to random X-chromosome inactivation^25,26^. Using OLIG2 and ARX double immunostaining, we examined if OLIG2 overexpression is detected in ARX positive or ARX negative cells (Fig. 2a). The anticipated mosaic pattern of ARX expression in the dorsal forebrain of the *Arx*^*cKO*/+^, revealed an inverse relationship between OLIG2 and ARX, suggesting that the presence of ARX in a cell results in repression of OLIG2 (Fig. 2a). It should be noted that this inverse relationship between ARX and OLIG2 is also detected in the ventral forebrain where expression domains of OLIG2 and ARX are mutually exclusive with the exception of a subset of cells at the boundary between the VZ and SVZ that co-express OLIG2 and ARX (Supplementary Fig. S1c). Together, these data suggest that ARX suppresses OLIG2 induction in the dorsal forebrain and to a lesser degree in the ventral forebrain during normal development.

To experimentally prove that ARX represses OLIG2 expression, an *Arx* expression construct was introduced into *Arx-*deficient brains by *in utero* electroporation (IUEP). OLIG2 was mostly eliminated from cells expressing exogenous *Arx* in the dorsal VZ of *Arx* cKO brains (Fig. 2b,c), confirming a repressive role for ARX in *Olig2* expression. Interestingly, we noted this repression was not observed in a subset of cells outside of the VZ (see marked area with * in Fig. 2b). One explanation for this observation is that ARX suppresses OLIG2 only in VZ progenitor cells but not cells that have exited the VZ.

### *Arx* cKO dorsal forebrain is partially ventralized

**Figure 3:**
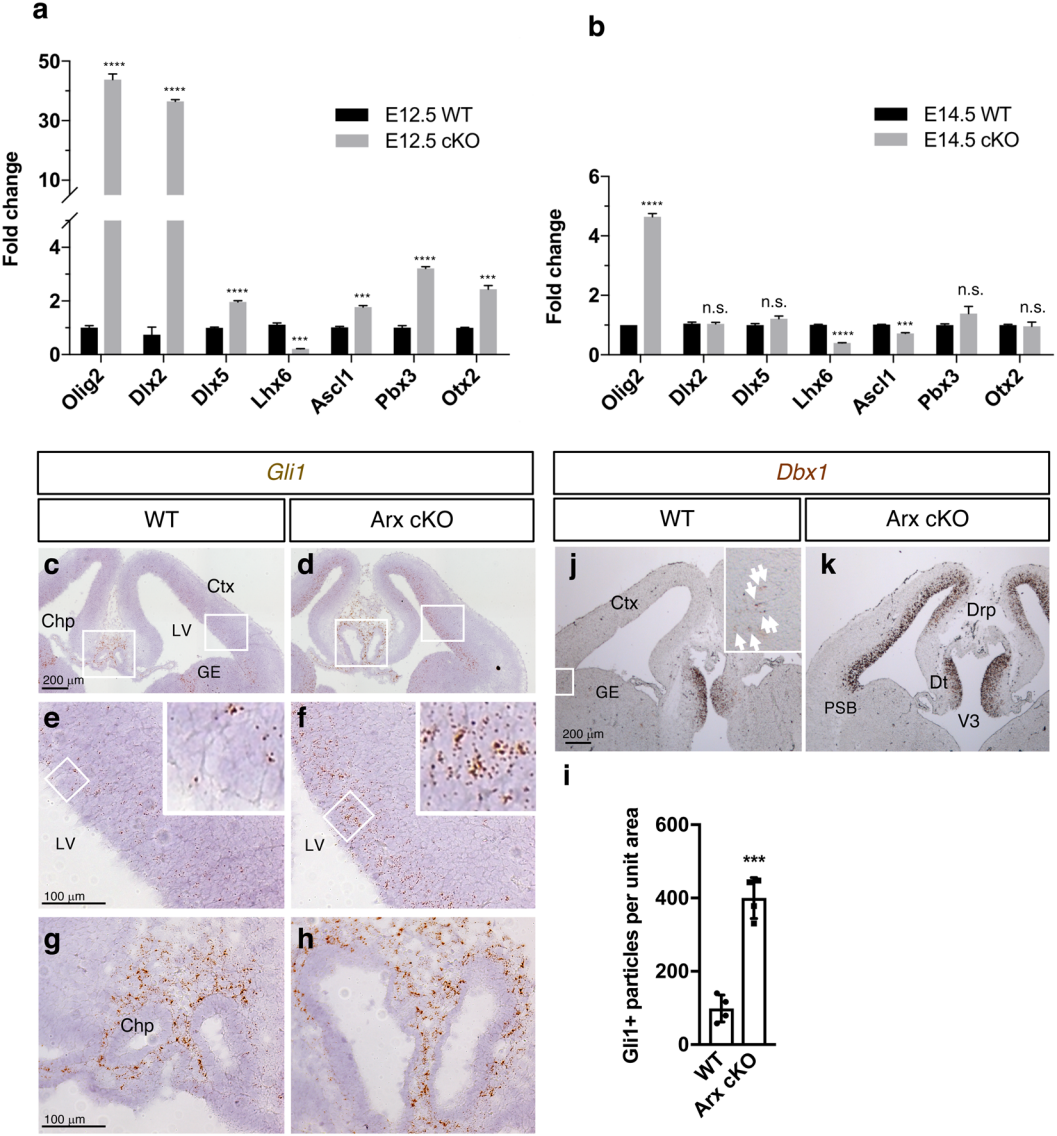
Dorsal forebrain is partially ventralized in *Arx* cKO mice. (**a**) Real-time quantitative PCR (qPCR) results for ventral forebrain markers in the embryonic cortices of E12.5 (**a**) and E14.5 (**b**) WT and *Arx*^*cKO/y*^ mice. Error bars: mean ± s.e.m (n = 3 per sample from two litters; ****P < 0.0001; ***P < 0.0005; *P = 0.0268; n.s., not significant; unpaired t-test). (**c**–**h**) Representative images of RNA *in situ* hybridization of *Gli1* (downstream target of SHH signaling) in WT (**c,e,g**) and *Arx*^*cKO/y*^ (**d,f,h**) embryonic brain (E13.5). (**e**,**f)** are magnified images of the smaller boxes in (**c**,**d)**, while (**g**,**h)** are magnified images of the bigger boxes in (**c**,**d)**. (**i)** Quantification of *Gli1* RNA *in situ* hybridization. Error bars: mean ± s.d (n = 4 sections for each genotype; ***P = 0.0002; unpaired t-test). (**j**–**k)**, Representative images of RNA *in situ* hybridization of *Dbx1*(marker for pallial-subpallial boundary, PSB) in WT (**j**) or *Arx*^*cKO/y*^ (**k**) embryonic brain (E13.5). White arrows in j indicate *Dbx1* positive cells in PSB. Insets in (**e,f,j)** are magnified images of the boxed areas. Ctx, neocortex; Drp, diencephalic roof plate; DT, dorsal thalamus; GE, ganglionic eminence; PSB, pallial-subpallial boundary; V3, third ventricle.

Given that *Olig2* normally shows strong expression in the developing ventral neural tube, we asked if the *Arx* cKO dorsal forebrain is abnormally ventralized. We closely re- examined the changes of the ventral genes in our previous *Arx*^−/*y*^ cortex microarray data (E14.5)^17^ and noticed a subset of upregulated ventral genes in the cortex (Supplementary Fig. S2a). Using RT-qPCR, we confirmed an up-regulation of a subset of the ventral genes (*Dlx2, Dlx5, Ascl1(Mash1), Pbx3 and Otx2*) in the *Arx* cKO cortex at E12.5 (Fig. 3a). Unlike *Olig2* that remained elevated (although at lower levels), these other genes no longer showed significant changes by E14.5 when compared to WT (Fig. 3b), suggesting the role for ARX in suppressing ventral gene expression is temporally restricted. We also found that *Dbx1*, whose expression is normally restricted to the pallial-subpallial boundary (PSB)^27^, is also ectopically expressed in the dorsal forebrain (Fig. 3d), further supporting an abnormal ventralization of the dorsal forebrain. Curiously, DLX2 protein, a ventral marker requiring high SHH activity for its induction^28^, was not detected despite its mRNA elevation (data not shown). Finally, we found small decreases in the level of expression for a subset of dorsal genes in the cortical progenitors including Pax6, Tbr2, Lhx2, Emx1/2, NeuroG2, and Dmrta1, when we re-examined our previous *Arx*^−/*y*^ cortex microarray data (E14.5)^17^ (Supplementary Fig. S2b). Reduction in PAX6 and TBR2 expression in protein level have been confirmed in our previous study^17^. Together, these data suggest dorsal progenitor cells are partially ventralized with a mixed identity.

### Activation of the SHH signaling pathway participates in the ectopic induction of *Olig2*

During embryonic development, SHH is known to induce ventral gene expression in the ventral neural tube^29^. *Olig2* is one of the genes induced at a low concentration of SHH^30^. Given the up-regulation of ventral genes including Olig2, we examined if SHH signaling was activated in the dorsal forebrain of *Arx* cKO mice. No *Shh* transcript was detected in the cortex of either WT or *Arx* cKO mice (E13.5), while its transcript was detected ventrally in both mice (Supplementary Fig. S2c), consistent with our previous microarray data^17^. Interestingly, *Gli1*, a SHH downstream target, was upregulated in the cortex of *Arx* cKO mice (Fig. 3c–i and Supplementary Fig. S3). Furthermore, both RT-qPCR and microarray assays demonstrate upregulation of *Ptch1* as well as *Gli1* in *Arx* mutant cortices, both SHH targets (Supplementary Fig. S3). Together these results suggest a possible upregulation of SHH signaling pathway, without changes in *Shh* transcript level itself, in *Arx* mutant cortices.

**Figure 4:**
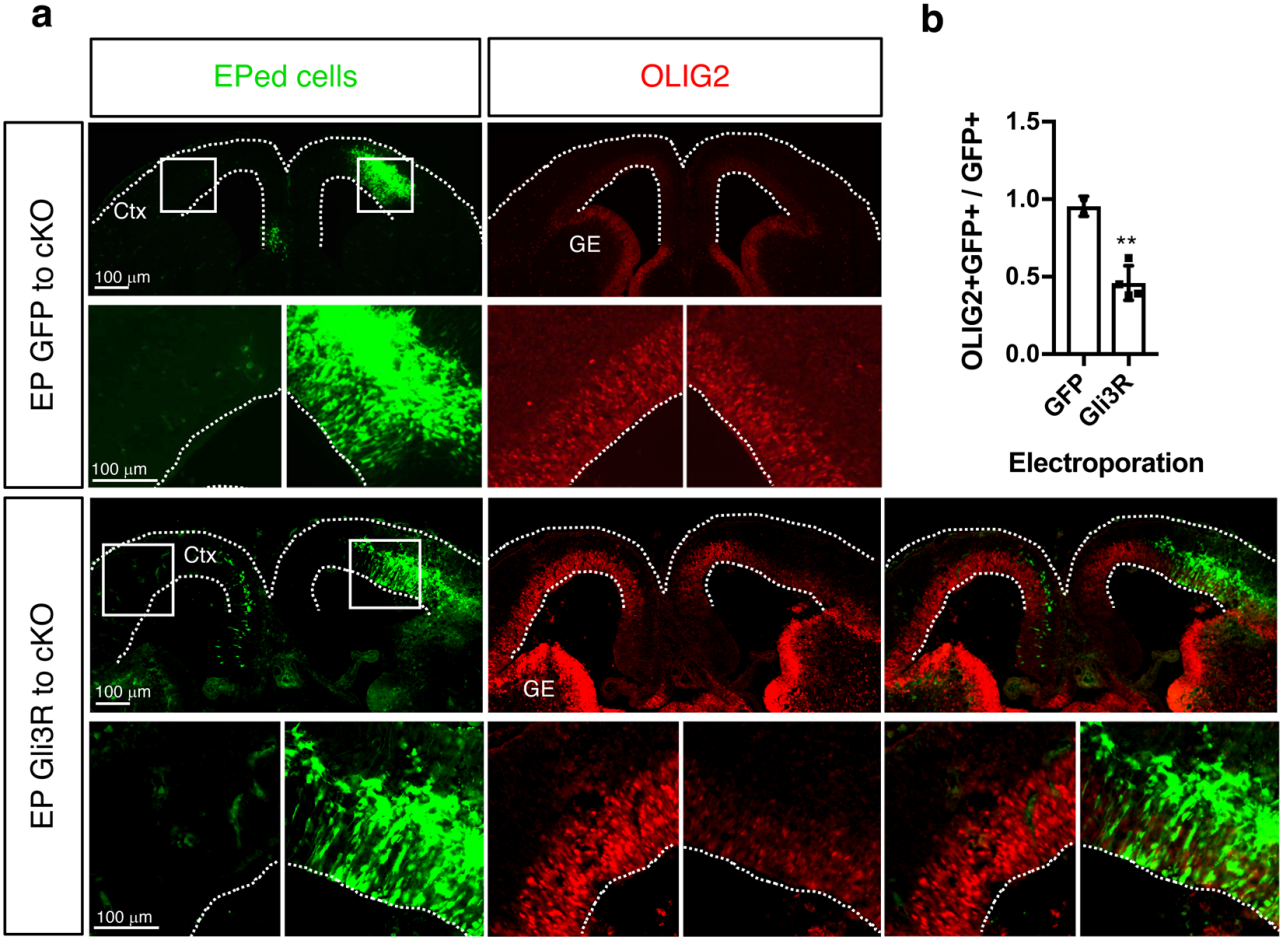
GLI3R can repress *Olig2* induction in *Arx*^*cKO/y*^ cortex. (**a**) Representative images of the E14.5 *Arx*^*cKO/y*^ cortex electroporated with a control GFP (*pCIG*)or Gli3R expression construct (*pCIG-Gli3R*) at E12.5 and double immunolabeled with GFP (green, EPed cells) and OLIG2 (red). The lower panels are magnified images of the boxed areas in the upper panels. Dotted lines mark the outlines of the sections. (**b**) Quantification of results in (**a**). The ratio of OLIG2+ GFP+ cells over GFP+ cells was plotted for both GFP and Gli3R electroporated samples. Error bars: mean ± s.d (n = 4 sections from two embryos per genotype; **P = 0.0034; unpaired t-test). CTX, embryonic neocortex; GE, ganglionic eminence.

To determine if SHH signaling is required for the abnormal *Olig2* induction in the *Arx* cKO cortex, we electroporated *Gli3R* (a Gli3 repressor construct to block SHH signaling)^31^ and analyzed OLIG2 expression. When *Gli3R* was electroporated to the *Arx* cKO cortex *in utero*, the ectopic OLIG2 overexpression was reduced (Fig. 4a,b), whereas a GFP expression construct did not change OLIG2 expression (Fig. 4a,b). These results support a role for the SHH pathway in the abnormal induction of *Olig2* and that blocking SHH signal is critical for normal cortical development.

### OLIG2 represses *Pax6* and *Tbr2* expression

We previously showed reduction in PAX6 as well as TBR2 expressing cells in cortical progenitors of *Arx* cKO mice^17^. A loss of PAX6 or TBR2 has been associated with a precocious cell cycle exit of intermediate progenitor cells (IPCs) and a disproportionate reduction in later-born, upper layer neurons, likely contributing to the small brains observed in mice mutant for these two genes^32,33^. These same phenotypes are observed in *Arx* cKO mice^17^. Interestingly, a transgenic mouse line with cortical *Olig2* overexpression also shares many findings with these mice^34^. These data suggest a common pathway involving *Arx, Olig2, Pax6* and *Tbr2* that results in proliferation/neurogenesis defects and small brains. During spinal cord development, it has been inferred that OLIG2 represses *Pax6* expression, although this was not directly tested^35^. Moreover, PAX6 is known to directly regulate *Tbr2* expression in the developing cortical IPCs^32^. We thus hypothesized that ectopically induced OLIG2 suppresses *Pax6* transcription and consequently *Tbr2*, and this suppression likely accounts for the reduction in PAX6 positive as well as TBR2 positive staining in *Arx* cKO mice^17^.

**Figure 5:**
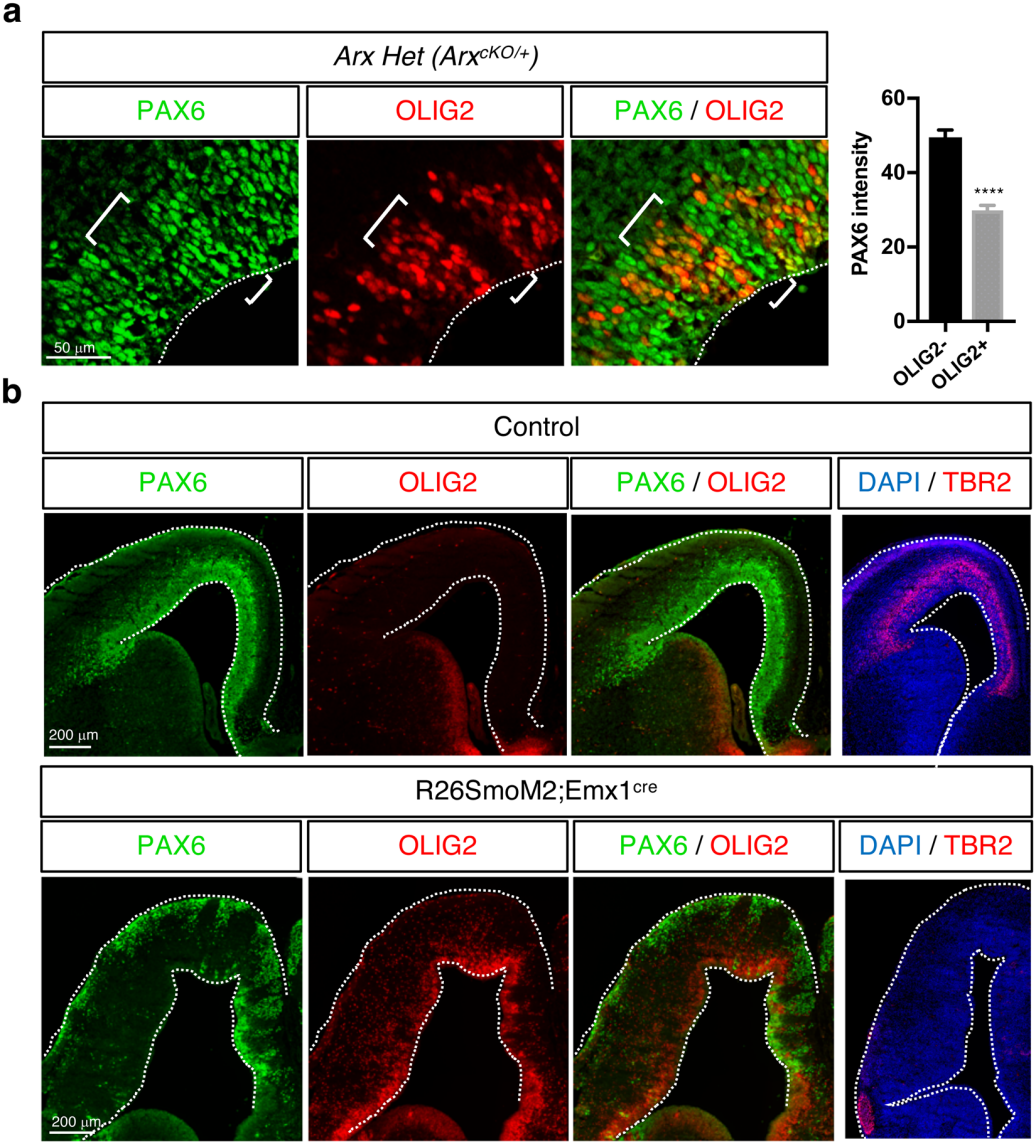
OLIG2 expressing cells have lower level of PAX6 and TBR2. (**a**) Representative images of PAX6 and OLIG2 double immunolabeling of *Arx* Het (*Arx*^*cKO*/+^) forebrain (E14.5). Longer brackets indicate areas with low PAX6 and high ARX, while shorter brackets mark areas with high PAX6 and no ARX. The quantification of PAX6 intensity in OLIG2+ or OLIG2- cells is shown in the graph. Error bars: mean ± s.e.m (n = 59 cells for WT; n = 153 cells for *Arx*^*cKO*/+^; ****P < 0.0001; unpaired t-test). (**b**) Representative images of PAX6 and OLIG2 double immunolabeling, or TBR2 immunolabeling of control or *R26SmoM2; Emx1*^*cre*^ embryonic cortex (E14.5).

To test this hypothesis, we took several approaches. First, we used *Arx* Het (*Arx*^*cKO*/+^) mice where WT and mutant cell columns, by virtue of random X-chromosome inactivation, are clonally distributed in the cortical VZ^25,26^. PAX6 expression levels were assayed in OLIG2 positive vs OLIG2 negative cells, taking advantage of the fact that OLIG2 positive cell columns (ARX negative) are clonally distributed adjacent to OLIG2 negative cell columns (ARX positive) in the VZ of the dorsal forebrain (see Fig. 5a). Lower levels of PAX6 were detected in OLIG2 positive cells (29.90 ± 1.301, n = 153) when compared to the adjacent OLIG2 negative cells (49.51 ± 1.945, n = 59) (Fig. 5a), supporting a repressive role of OLIG2 in PAX6 expression.

Second, we examined PAX6 and TBR2 expression in control and *R26SmoM2; Emx1*^*cre*^ mice (Fig. 5b) which have abnormal OLIG2 induction in the dorsal forebrain due to constitutively activated SHH signaling^36^. OLIG2 positive cells in the VZ of the dorsal forebrain show little or no PAX6 expression and PAX6 positive cells are located where OLIG2 is not present (Fig. 5b), also suggesting repression of PAX6 expression by OLIG2. We also observed almost complete loss of TBR2 expression in the dorsal VZ of *R26SmoM2; Emx1*^*cre*^ mice (Fig. 5b) when compared to controls (Fig. 5b).

**Figure 6:**
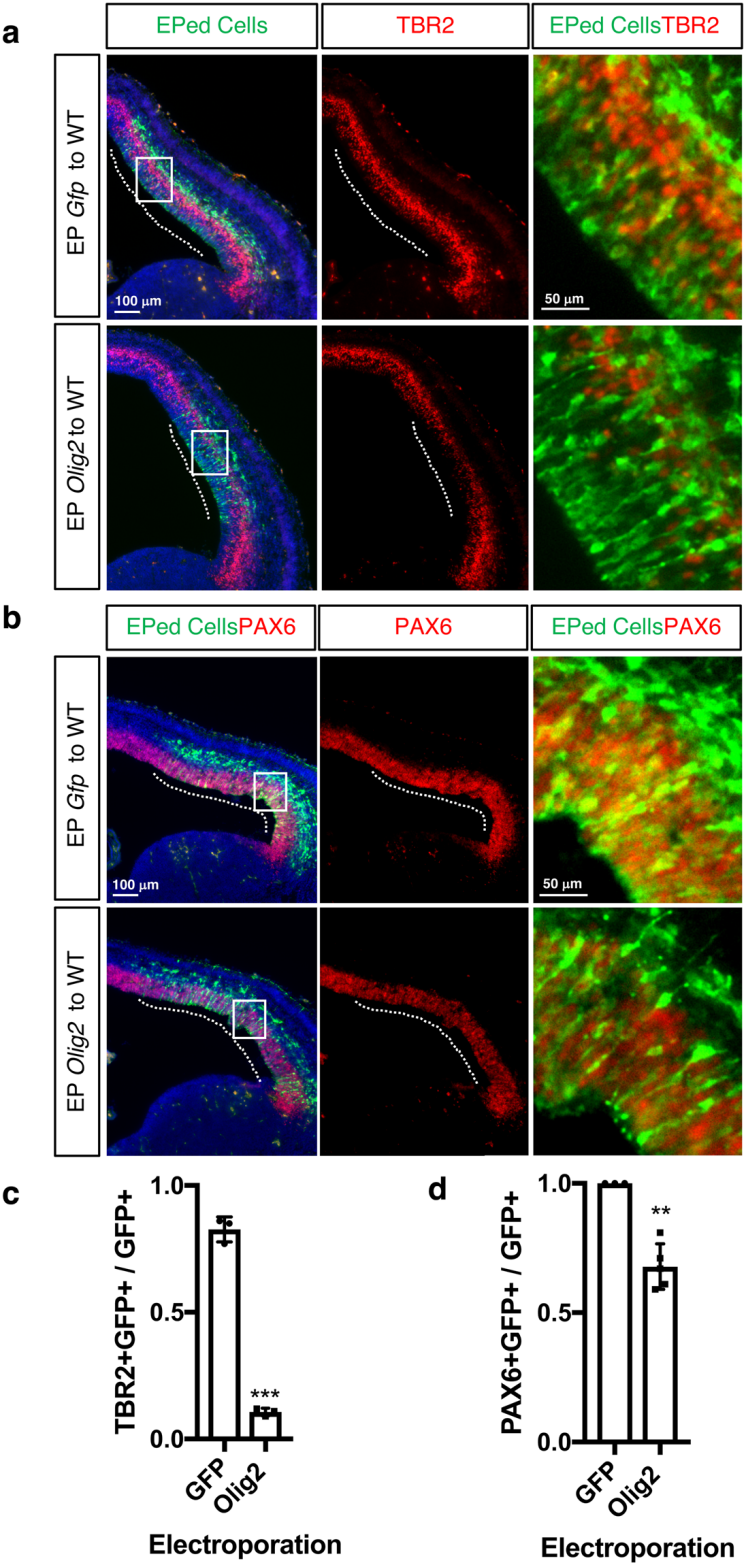
Forced expression of *Olig2* represses *Pax6* and *Tbr2* expression. (**a,b)** Representative images of the immunofluorescent labeling of the WT embryonic cortex electroporated with a control GFP (*pCIG*) or *Olig2* expression construct (*pCIG-Olig2*) (E13.5 → E14.5). Antibodies against GFP (green) and TBR2 (red) (**a**) or PAX6 (red) (**b**) were used. The number of PAX6+ and TBR2+ cells are reduced in the *Olig2* electroporated cortices compared to the control. (**c,d)** Quantification of results in a (**c**) and b (**d**). The ratio of TBR2+ GFP+ or PAX6+ GFP+ cells over GFP+ cells was plotted for GFP or Olig2 electroporated brains. Error bars: mean ± s.d (For Tbr2, n = 3 for each sample; ***P = 0.0007; For Pax6, n = 3 for GFP; n = 5 for Olig2; **P = 0.0012; unpaired t-test).

Third, we tested if forced expression of *Olig2* can repress *Pax6* and *Tbr2*. Electroporation of a GFP-tagged empty vector (pCIG) or GFP-tagged *Olig2* expression construct (pCIG-*Olig2*) into the cortex of WT mice (E13.5) showed that both PAX6 (Fig. 6b) and TBR2 (Fig. 6a) expression were reduced in *Olig2* electroporated cortices (PAX6: 0.68 ± 0.039, n = 5)(TBR2: 0.11 ± 0.009, n = 3)(harvested at E14.5) compared to control electroporated cortices (PAX6: 1.00 ± 0.000, n = 3)(TBR2: 0.83 ± 0.028, n = 3), although the reduction of TBR2 was greater than that observed with PAX6 (Fig. 6c,d). Given that PAX6 has been shown to positively regulate *Tbr2* expression^32^, we postulate that the TBR2 reduction could be explained by reduced *Pax6* expression.

**Figure 7:**
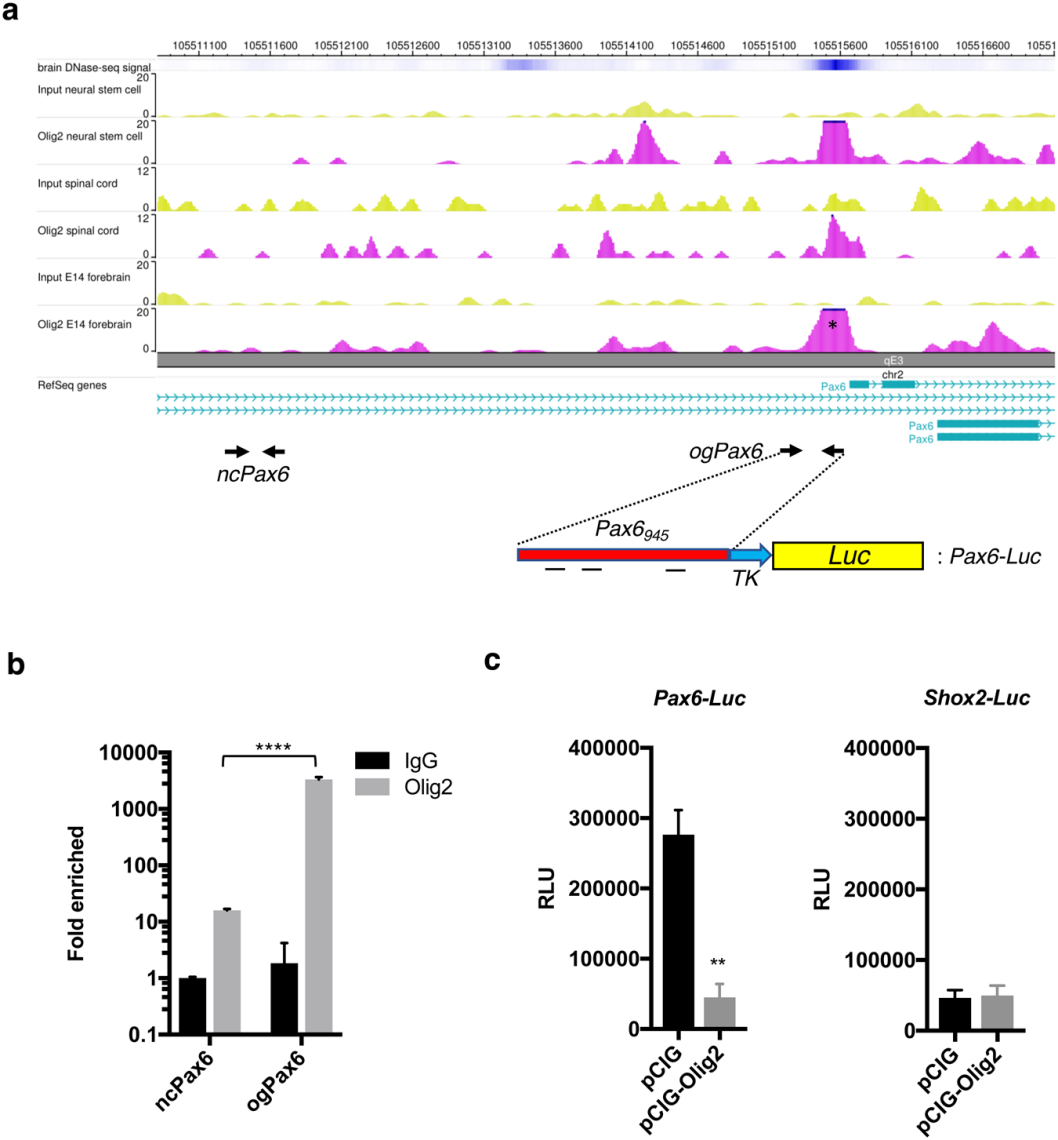
OLIG2 directly represses *Pax6* transcription. (**a**) OLIG2-ChIP-seq assay identified putative OLIG2 binding region (pink, marked with *) in *Pax6* genomic sequence upstream to transcription start site. Arrows labeled as *ncPax6* and *ogPax6* indicate the locations of two primer pairs, *ncPax6* (negative control primers away from OLIG2 binding region) and *ogPax6* (experimental primers from OLIG2 binding region), respectively, used for OLIG2-ChIP-qPCR in b. The schematic diagram below the arrow depicts *Pax6-Luc* reporter construct used in c. Within the 945 bp *Pax6* genomic sequence (red, Pax6_945_), there are three putative consensus bHLH TF binding sites. (**b**) ChIP-qPCR results using two different proteins, IgG and OLIG2, for ChIP experiment, and two different primer sets, *ncPax6* and *ogPax6*, for subsequent qPCR. Error bars: mean ± s.d (n = 3; ****P < 0.0001; unpaired t-test). IgG and *ncPax6* were used for negative control. (**c**) Quantification of reporter gene assays testing the effects of OLIG2 (pCIG-*Olig2*) and GFP (pCIG, control) on *Pax6-Luc* and *Shox2a-Luc* (control) reporter constructs. Error bars: mean ± s.d (n = 3; **P = 0.0027; unpaired t-test).

Finally, we investigated if OLIG2 can directly regulate *Pax6* expression at the level of transcription. For this, we first performed a ChIP-seq assay using an OLIG2 antibody and E14.5 embryonic forebrain. Our OLIG2-ChIP-seq data identified putative OLIG2 binding sites in the upstream *Pax6* genomic sequence (Fig. 7a), which are consistent with previously identified sequences in the spinal cord and neural progenitor cells from embryonic mouse stem cells (ref.^37^; publicly available data, see methods). Next, to validate these binding sites, we performed two independent experiments; ChIP-qPCR and reporter gene assays. For ChIP-qPCR, ChIP was conducted with an OLIG2 antibody as well as IgG, followed by qPCR with the primer sets, *ogPax6*, for the Pax6 genomic sequence identified in OLIG2 ChIP-seq (chr2:105515511-105515700 on mm9) as well as *ncPax6*, for the Pax6 genomic sequence distal to the identified sequence as a negative control (Fig. 7b). A dramatic enrichment of the putative OLIG2 binding sites were detected when compared to negative control sequences, when OLIG2-ChIPed DNAs were used (3,323 ± 198, n = 3 vs 15.96 ± 0.504, n = 3). These data validate our ChIP-seq identified sequences (Fig. 7b). Next, a *Pax6-Luc* reporter construct was generated, which contains *Pax6* genomic sequence (*Pax6*_*945bp*_ including ChIP-seq-identified *Pax6* sequence) as an upstream promoter driving luciferase expression, in addition to the herpes virus thymidine kinase minimal promoter (Fig. 7a). Reporter gene assays were conducted using *Pax6-Luc* construct as well as *Shox2*-*Luc* construct, negative control, which contains *Shox2a* promoter sequence instead (*Shox2a* is an ARX target; ref.^38^) (Fig. 7c). Upon co-transfection, an OLIG2 expression construct (pCIG-Olig2) significantly down-regulated *Pax6-Luc* reporter activity when compared to a control construct (pCIG) (45,160 ± 19,007, n = 4 vs 276,615 ± 34,939, n = 4), demonstrating that the ChIP-identified *Pax6* genomic sequences act as a transcription regulatory element responsive to OLIG2. In contrast, an OLIG2 expression construct did not change *Shox2*-*Luc* reporter activity (49,815 ± 14,009, n = 4 vs 46,490 ± 10,874, n = 4) (Fig. 7c), supporting OLIG2-mediated repression being specific to the *Pax6* promoter. These results strongly suggest that OLIG2 can repress *Pax6* expression at the level of transcription. In contrast, we did not find *Tbr2* regulatory sequences in our OLIG2-ChIP-seq data, implying that *Tbr2* transcription may not be directly regulated by OLIG2.

Collectively, our data presented here provide evidence to support our postulate that the ectopic induction of *Olig2* in *Arx* cKO forebrain represses *Pax6*, which leads to a reduction in *Tbr2* expression, likely contributing to the microcephaly phenotype observed in these mice and patients with *ARX* mutations^13–17^. These findings support a model wherein OLIG2 expression is actively repressed by ARX during normal development in the dorsal forebrain, permitting PAX6 and TBR2 expression. Therefore, our data implicate a role for ARX in modulating brain size through regulating *Olig2-Pax6-Tbr2* pathway. Previously we have shown another mechanism whereby ARX participates in brain size control; ARX regulates *Cdkn1c* transcription and the upregulation of *Cdkn1c* in *Arx* cKO mice, which results in premature cell cycle exit of progenitors, is likely cause of decrease in IPC population, eventually small brain. Thus, we tested whether OLIG2 overexpression would influence *Cdkn1c* expression. Our data demonstrate that upon *Olig2* overexpression in WT mice, *Cdkn1c* expression does not change (Supplementary Fig. S4). These data suggest that these two pathways do not converge but act in parallel. Taken together, our data indicate that ARX participates in cortical size control through at least two different mechanisms: by regulating *Olig2*-*Pax6*-*Tbr2* pathway and/or by repressing *Cdkn1c* transcription.

## Discussion

Disorders of brain development commonly include multiple and complex phenotypes that cannot be explained by perturbation in a single process (e.g. cell proliferation). Brain abnormalities associated with *ARX* mutations are an excellent example; patients with *ARX* mutations show brain size and structure anomalies, which suggest disruptions in more than one process. Our data provide insight into how mutations in one gene, *ARX*, can disrupt both brain growth and patterning. In mice where *Arx* has been conditionally abrogated from the cerebral cortex, we found ventral genes, such as *Olig2*, are abnormally expressed dorsally. Furthermore, the ectopically expressed OLIG2 represses dorsal specific PAX6 and TBR2 expression, both important for cortical progenitor proliferation^32,33^. Thus, our current findings together with our previous work^17^ identify ARX as a critical transcription factor impacting DV specification as well as proliferation of cortical progenitor cells.

OLIG2 is a basic helix-loop-helix (bHLH) transcription factor that is known to have essential roles in cell fate specification and cell proliferation^21–24^. During early spinal cord development, OLIG2 first specifies motor neuron precursors and then promotes their cell cycle exit and neuronal differentiation^39,40^. Later in spinal cord development, OLIG2 directs the formation of oligodendrocyte precursors and mature oligodendrocytes^41,42^. In the developing forebrain, OLIG2 is expressed in progenitor cells of the GE (with the highest levels of expression in the MGE domain) that give rise to subtypes of cortical interneurons as well as oligodendrocytes^43,44^. We found no detectable changes in the number of oligodendrocytes as well as no detectable changes in cell death in the *Arx* cKO brain (data not shown). These data demonstrate that the enhanced OLIG2 expression does not contribute to the generation of additional cells of the oligodendrocyte lineage in the mature brain. A recent study in an *Olig2* transgenic mouse showed OLIG2 overexpression inhibits cortical progenitor proliferation and neurogenesis, leading to a severe reduction in brain size and a disruption in cortical lamination^34^. These brain phenotypes are similar to what we observe in *Arx* cKO mice which also overexpresses *Olig2*. Given that *OLIG2* maps to the Down’s syndrome critical regions on chromosome 21, these data support a provocative model for a potential role of *OLIG2* in the developmental brain defects associated with Down syndrome such as intellectual disability. In this syndrome *OLIG2* is triplicated and over-expressed^45^, suggesting a common pathway resulting in intellectual disabilities as well as microcephaly in patients with Trisomy 21 and *ARX* mutations.

During development *Olig2* is known to be induced by low levels of SHH signaling in the ventral spinal cord and brain^35,46^. Our *Gli1* RNA *in situ* hybridization data support that SHH signaling activity might be present, although weak, during normal cortical development^47^. Another support for this comes from our observation of a weak OLIG2 expression in dorsal forebrain progenitor cells, prior to the arrival of migrating OLIG2 positive cells from the GE, which has not been previously reported (Fig. 1). With the loss of ARX, both *Gli1* and *Olig2* expressions become strongly activated, suggesting an elevated SHH signaling activity. Although the role of SHH in ventral specification of the forebrain is well established, the role of SHH signaling in cerebral cortical development is less clear, as the source of SHH protein is uncertain^6,48^. There are at least two potential SHH sources for cortical development; the cerebrospinal fluid (CSF) and the developing cortex itself^49,50^. SHH levels peak in the CSF at E10.5 and fall to low levels by E14.5^49,50^. Our RNA *in situ* hybridization data suggest that *Shh* transcript is not present or in very low levels (undetectable) in the developing cortex (E14.5) (Supplementary Fig. S2). Moreover, our data provide indirect support for CSF being a SHH source that influences cortical development, as the overexpression of ventral markers in *Arx* cKO coincides with this timing of SHH exposure; their upregulation was observed at E12.5 but not E14.5, except for *Olig2* which was still detected at E14.5 but at lower levels, consistent with the known inducibility of *Olig2* even at low levels of SHH signaling^35,36^. One possible role for ARX in the dorsal forebrain is to suppress *Olig2* induction either by directly repressing *Olig2* transcription or by indirectly repressing SHH signaling that can induce *Olig2*, or a combination of the two. Since we did not find *Olig2* regulatory sequences in previously published ARX ChIP-on-ChIP data^51^, ARX appears not to regulate *Olig2* transcription directly. However, we cannot rule out the possibility that ARX could bind to the *Olig2* enhancer region and regulate its transcription from a distant location, since this study only interrogated the promoter region. Further studies are required to elucidate the specific mechanism by which ARX suppresses *Olig2* induction during normal cortical development.

The enhanced expression of *Olig2* at an early stage of neurogenesis in the *Arx* cKO likely has a significant impact on neurogenesis. In addition to the *Cdkn1c* overexpression as we previously reported^17^, reduced PAX6 and TBR2 expression likely accounts for some of the neurogenesis defects reported in the *Arx* mutant mice and the associated microcephaly^17^. Given that *Cdkn1c* expression does not change upon *Olig*2 electroporation, it appears that *Cdkn1c* upregulation in *Arx* cKO is independent from OLIG2 overexpression. PAX6 is essential for controlling the balance between neural stem cell self-renewal and neurogenesis^32,52^. Altering the levels of PAX6, either up or down, leads to a small brain through different mechanisms: increasing PAX6 levels drives the system towards neurogenesis, while removing *Pax6* reduces cortical stem cell self-renewal^32^. TBR2 is expressed in the IPCs and can direct conversion of RGs into IPCs^53^. Its loss in the developing forebrain results in the loss of IPCs leading to a smaller brain phenotype^33^. Although OLIG2 seems to directly regulate *Pax6* transcription as shown with our OLIG2 ChIP-seq data, *Tbr2* transcription does not appear to be directly regulated by OLIG2 since *Tbr2* regulatory sequences were not detected in our ChIP-seq analysis. We postulate that the reduction in TBR2+ cells in *Arx* cKO could be explained by combined effects of 1) reduced PAX6 by OLIG2 overexpression leading to *Tbr2* repression, and 2) upregulated *Cdkn1c l*eading to premature cell cycle exit thus reducing the TBR2+ IPC population. Given the roles of these two genes in RGs and IPCs for cortical neurogenesis, it is reasonable to consider that their repression, due to OLIG2 overexpression, accounts for at least a part of the neurogenesis defects observed in the *Arx* cKO mice.

In summary, the misregulation of OLIG2 in the *Arx* cKO cortex and its relationship with PAX6 and TBR2 repression, link the control of cellular identity to the regulation of the cortical size. Our data also indicate ARX plays a critical role in coordinating dorsal progenitor cell proliferation and specification in the mammalian forebrain. Furthermore, our data implicate early patterning defects as components in the pathogenesis of the developmental anomalies and neurological phenotypes associated with *ARX* mutations in human patients.

## Materials and Methods

### Animals

All animal experiments were performed in accordance with the relevant guidelines and regulations approved by the Harvard Medical School Institutional Animal Care and Use Committee (protocol no. 04946), and Brigham and Women’s Hospital Institutional Animal Care and Use Committee (protocol no. 2016N000244). *CD1* mice (Stock No. 022) were purchased from Charles River Laboratories, and the *Emx1*^*cre*^ (Stock No. 005628) and R26 SmoM2 (Stock No. 008831) mice from The Jackson Laboratory. The floxed *Arx* (*Arx*^*flox*^) mice were bred and maintained on a C57BL/6 background as previously described^38^. Dorsal telencephalic mutant *Arx*^*flox/y*^*; Emx*^*Cre*^ (subsequently referred to as *Arx*^*cKO/y*^ or *Arx* cKO) mice were generated by mating *Arx*^*flox*/+^ or *Arx*^*flox/flox*^ females to *Emx1*^*cre*^ males*, and R26SmoM2; Emx1*^cre^ mice were generated by mating R26 *SmoM2 (homozygote)* females to *Emx1*^*cre*^ males. As *Arx* is an X-chromosome gene, we used only male conditional knock out (*Arx*^*cKO/y*^) and male wild type (*Arx*^+/*y*^) mice for consistent comparison unless noted otherwise.

### *In utero* electroporation (IUEP)

*In utero* electroporation (IUEP) was performed at embryonic day 12.5 (E12.5) or E13.5 as described previously^54^. One or two days after EP dams were sacrificed, and the brains were removed for analysis. For quantification, GFP+ electroporated cells were manually marked and counted as individual ROI, and the mean intensity of OLIG2, PAX6, or TBR2 in these ROI were measured in Image J. If the intensity of these staining in the particular ROI was higher than the background, those ROI were counted as double positive cells. At least three embryos from two or three separate electroporation were analyzed for each condition.

### DNA constructs

The pCAG-IRES*-Gfp* (pCIG) was used as a control for IUEP. The pCAG*-Arx-*IRES*-Gfp* (pCIG*-Arx*) was described previously^55^. To generate pCIG*-Olig2*, full length cDNAs encoding *Olig2* (NM_016967) was derived by PCR from mouse cDNAs generated from the total RNA extracted from E12.5 brain lysates (see Table 1 for primer sequences). These PCR products were cloned into pCIG vector (EcoRI and MluI sites of pCIG*-Arx*) and replaced *Arx* cDNA sequences, using GeneArt Seamless Cloning and Assembly kit (Life Technologies). pCAG*-Gli3R-*IRES*-dsRed* was generated by inserting PCR product containing 1–645 amino acid residues of GLI3 to EcoRI and MluI sites of pCAG*-*IRES*-Gfp* and by replacing GFP sequence with dsRed coding sequence. To generate the *Pax6-Luc* reporter construct, the 925 bp Pax6 genomic sequence (chr2:105515322-105516246) containing a putative OLIG2 binding region identified in OLIG2 ChIP-seq (chr2:105515511-105515700 on mm9), was PCR amplified from genomic DNA and cloned into *Bam*HI and *Hind*III sites of the empty *TK-Luc* construct (*MCS-TK-Luc*) that carries the herpes virus thymidine kinase minimal promoter (−105/+51) and luciferase coding sequence^38^. The *Shox2-Luc* reporter gene construct has been described previously^38^.

**Table 1:** Primer sequences used in this study.

Subcloning	Forward primer (F-5′ to 3′) Reverse primer (R-5′ to 3′)
Gli3R	F-CATCATTTTGGCAAAGCACCATGGAGGCCCAGGCCCAC AGCTCTACGG R-AGAAGCTTCTGCAGAACTAGTCCCCACGCTGCTTCTTGGTAACA
Olig2	F-TCGAGCTCAAGCTTCGCACCATGGACTCGGACGCCAGCCTGG R-TAGAAGCTTCTGCAGATTTCACTTGGCGTCGGAGGTGAGGC
**RT-qPCR**	**Forward primer (F-5′ to 3′)** **Reverse primer (R-5′ to 3′)**
Olig2	F-CAAATCTAATTCACATTCGGAAGGTTG R-GACGATGGGCGACTAGACACC
Dlx2	F-GTCTCCTACTCCGCCAAAAGC R-GGATTTCAGGCTCAAGGTCTTCC
Dlx5	F-CAACTCAGTGGAGCATTCCGAC R-GGCTTTGCTCTCGAAGGAGGTT
Lhx6	F-CGTTGAGGAGAAGGTGCTTTGC R-GCTTGGGCTGACTGTCCTGTTC
Ascl1	F-CGGAACTGATGCGCTGCAAACG R-GGCAAAACCCAGGTTGACCAAC
Nr2f1	F-CCAACAGGAACTGTCCCATCGA R-CCGTTTGTGAGTGCATACTGGC
Pbx3	F-TCGGAGCCAATGTGCAGTCACA R-TTGCGTCCTGCCAGCCTCCAT
Otx2	F-TGAGGGAAGAGGTGGCACTGAA R-GCCTCACTTTGTTCTGACCTCC
Ptch1	F-CCTCGCTTACAAACTCCTGGTG R-TGATGCCATCTGCGTCTACCAG
Gli1	F-CTCAAACTGCCCAGCTTAACCC R-TGCGGCTGACTGTGTAAGCAGA
Gapdh	F-ATCTTCTTGTGCAGTGCCAGCCTCGTCCCG R-AGTTGAGGTCAATGAAGGGGTCGTTGATGG
Cdkn1c	F-AGCTGAAGGACCAGCCTCTCTC R-ACGTCGTTCGACGCCTTGTTCT
**Chip-qPCR**	**Forward primer (F-5′ to 3′)** **Reverse primer (R-5′ to 3′)**
ncPax6	F-GAACTTGGACTTCAGGCAGGAC R-CTGGAGGACCTAAGGCACTGG
ogPax6	F-CGGCAGAGCCGAAAACAAGTG R-TCATCCTCCAGCAAAACACTTCCTC

### Immunohistochemistry (IHC), immunofluorescent (IF) labeling and quantification

Embryonic mouse brains were fixed overnight in 4% paraformaldehyde and processed for cryosections (15 μm) as previously described^55^. Control and experimental sections were collected on the same slide. IHC and IF labeling were performed using previously described protocols^55^. Primary antibodies used in this study included rabbit monoclonal anti-OLIG2 (1:100, Abcam, ab109186), mouse monoclonal anti-OLIG2 (1:100, Millipore Sigma, MABN50), chicken polyclonal anti-GFP (1:500, Invitrogen, A10262), rabbit polyclonal anti-RFP (1:500, MBL International, PM005), rabbit polyclonal anti-ARX (1: 100, Dr Kitamura), rabbit polyclonal anti-PAX6 (1:1000, Covance, PRB-278P), and rabbit polyclonal anti-TBR2 (1:200, Abcam AB15894 or a gift from Dr. Robert Hevner, Seattle Children’s Hospital). Appropriate secondary antibodies either biotinylated (1:500, Vector Lab) or conjugated with a fluorescent dye (Alexa-Fluor 488 or Alexa-Fluor 594; 1:200; Invitrogen) were used for IHC or IF, respectively. Tyramide amplification (Invitrogen) was used for mouse anti-OLIG2. AB reagents (Vector Laboratories) and 3,3′-Diaminobenzidine (DAB) (Vector Laboratories) were used as recommended by manufacturer to detect the signals in IHC. IF nuclear labeling was with 4’,6-diamidino-2-phenylindole (DAPI, Molecular Probes). Light microscopy images were captured on an Olympus BX43 microscope equipped with an Olympus DP26 camera using Cell Sens software, or Nikon eclipse E400 microscope with Leica DFC 420 camera using LAS AF Lite software (version 2.6.3).

Immunofluorescent images were captured on Zeiss Observer Z1 inverted microscope equipped with a Hamamatsu ORCA-Flash4.0 camera using Zeiss Zen Pro software. For some fluorescent images, multiple tiled images were taken at 20x and stitched using Zeiss Zen Pro software. When necessary, entire image level and brightness were adjusted with Adobe Photoshop CS5, or Image J (version 2.0.0). For PAX6 intensity quantification, DAPI staining was used to mark each cell as individual ROI, and the mean intensity of OLIG2 and PAX6 in each ROI was measured in Image J. OLIG2+ cells were called if the mean intensity was higher than background.

### mRNA *in situ* hybridization and quantification

Embryonic mouse brains were fixed overnight in 4% paraformaldehyde and processed for cryosections (15μm) as previously described^55^. Control and experimental sections were collected on the same slide. To detect mouse *Dbx1*, *Gli1*, and *Shh* mRNAs, RNAscope® 2.5 HD detection kit (brown) (ACDBio) was used following manufacturer’s recommendation. For quantification, four serial sections from each genotype were imaged (Nikon eclipse E400 microscope with Leica DFC 420 camera using LAS AF Lite software (version 2.6.3)) and *Gli1* positive granules within 200 μm wide area spanning the entire neural tube (from ventricular surface to pial surface) taken from the middle part of the section (see Supplementary Fig. S3a’–f’ for examples) were automatically counted using Image J (Images underwent thresholding using Otsu method, cut off value 243). Once quantification was completed, the slides were stained with diluted Hematoxylin for weak counterstaining and imaged again.

### Reverse transcription quantitative real time PCR (RT-qPCR)

Total RNA was isolated using the RNeasy plus kit (Qiagen, CA, USA) from E12.5 or E14.5 neocortices. For *Cdkn1c* RT-qPCR, total RNA was isolated from E14.5 neocortices that were *in utero* electroporated with pCIG-*Olig2* or pCIG at E13.5. cDNA was synthesized by High-Capacity cDNA Reverse Transcription Kit (Applied Biosystems, using random hexamers) according to the manufacturer’s instructions. RT-qPCR was performed in StepOne Plus Real Time PCR System (AB applied biosystems) using SYBR green PCR master mix (AB applied biosystems) in 20 μl reaction volume in triplicate. The reaction was done as following: 95 °C for 10 min, 95 °C for 15 sec, and 60 °C for 1 min, 40 cycles. Primers used are listed in Table 1. Cycle threshold (CT) values were normalized by glyceraldehyde-3-phosphate dehydrogenase (*Gapdh*).

### Chromatin immunoprecipitation-sequencing (ChIP-seq) procedure

Embryonic dorsal forebrains (E14.5) were triturated in Hank’s Balanced Salt Solution (Thermo Fisher Scientific) and fixed in 1% fresh paraformaldehyde in PBS for 5 min at room temperature. Fixation was quenched with 125 mM Glycine at room temperature for 5 min and washed with cold PBS twice. Chromatin immunoprecipitation was performed using MAGnify Chromatin immunoprecipitation system (Thermo Fisher Scientific) with minor modifications as follows. Chromatin was fragmented into 100–300 bp by setting the Bioruptor UCD-200 (Diagenode) to high power and sonicated for 3 rounds of 10 cycles (30 sec ON/30 sec OFF). For immunoprecipitation of Olig2-bound chromatin, 2 μg of anti-Olig2 antibody [EPR2673] (Abcam, ab109186) was incubated with cleared chromatin lysate and 2 μg of whole rabbit IgG was used as a control. Input and ChIPed DNA libraries were prepared using an Illumina Next Seq (single-end reads of 75 bp).

### ChIP-seq data analysis

FASTQ sequences were aligned to the mouse mm9 genome sequence using HISAT2^56^ and converted to SAM and then BAM files. Then, ChIP-seq peaks were called using MACS2^57^, input DNA without ChIP as reference, and with the default settings. External data (GSE103324 and GSE74646) were analyzed by the same settings previously used^37^. The HISAT2-aligned peaks and MACS-determined peak positions were visualized using WASHU Epi Genome Browser.

### Chromatin immunoprecipitation-quantitative real time PCR (ChIP-qPCR)

Control IgG- and OLIG2-ChIPed DNA libraries were prepared as described above in ChIP-seq procedure and used for qPCR. StepOne Plus Real Time PCR System (AB applied biosystems) using SYBR green PCR master mix (AB applied biosystems) in 20 μl reaction volume in triplicate was used for qPCR. Following the same procedure described above in the RT-qPCR methods, qPCR was performed with a negative control primer pair (cPax6F1and cPax6R1) whose sequences are from the Pax6 genomic region (chr2:105512527-105512827) not associated with OLIG2 binding, and an experimental pair (oPax6F1 and oPax6R1) whose sequences are from the Pax6 genomic region (chr2:105515511-105515700 on mm9) identified in our OLIG2-ChIP-seq as well as previously published^37^ or publicly available OLIG2-ChIP-seq data (GSE103324 and GSE74646). All reactions were performed in triplicate. Cycle threshold (CT) values were normalized to IgG control.

### Reporter gene assay

*Pax6-Luc* or *Shox2-Luc* was co-transfected into HEK293T cells with p*CIG* or p*CIG-Olig2*. Twenty-four hours later luciferase activity was measured with a POLARstar Omega microplate reader (BMG LABTECH, Ortenberg, Germany) as previously described^58^.

### Experimental Design and Statistical Analysis

All IUEP experiments were repeated at least three times with three different litters. Each image for IUEP analysis was taken from representative images of the sections from at least three brains from two or three different litters (average 10 sections per brain). All IUEP were performed with appropriate controls such as empty vector expressing GFP, or compared with un-electroporated control side. All immunostaining or mRNA *in situ* hybridization on embryonic brain sections were also performed with appropriate controls such as wild type brain (only male mice were used for comparison between Arx cKO and WT) or Cre negative brain sections. All statistical analyses were done in Prism software using 2-tailed unpaired Student’s t-test (with Welch’s correction). All graphs are plotted as mean ± the standard deviation (s.d) or mean ± the standard error of the mean (s.e.m).

## Electronic supplementary material

Supplementary Information
