# Supplementary material for: Arx Expression Suppresses Ventralization of the Developing Dorsal Forebrain
Source: Sci Rep. 2019 Jan 18;9:226. doi: 10.1038/s41598-018-36194-6 (PMC6338776; doi:10.1038/s41598-018-36194-6)
Supplement: Supplementary file 1 — Supplementary Information [file 41598_2018_36194_MOESM1_ESM.pdf]

Arx Expression Suppresses Ventralization of the Developing Dorsal Forebrain

Youngshin Lim[1#], Il-Taeg Cho[1#], Xiuyu Shi[1,2], Judith B. Grinspan[3], Ginam Cho[1*] and Jeffrey A. Golden[1*]

[1]Department of Pathology, Brigham and Women's Hospital, Harvard Medical School, Boston, MA 02115; [2]School of Life Sciences, Xiamen University, Xiamen, Fujian 361005, China; [3]Department of Neurology, Children's Hospital of Philadelphia, University of Pennsylvania Perelman School of Medicine, Philadelphia, PA 19104

[#] These authors contributed equally to this work.

[*] Corresponding authors:

Ginam Cho (gpcho@bwh.harvard.edu)

Jeffrey Golden (jagolden@bwh.harvard.edu)

Department of Pathology

Brigham and Women's Hospital

Harvard Medical School

75 Francis Street

Boston, MA 02115

Ectopic OLIG2 in *Arx* cKO indicates that ARX suppresses *Olig2* expression

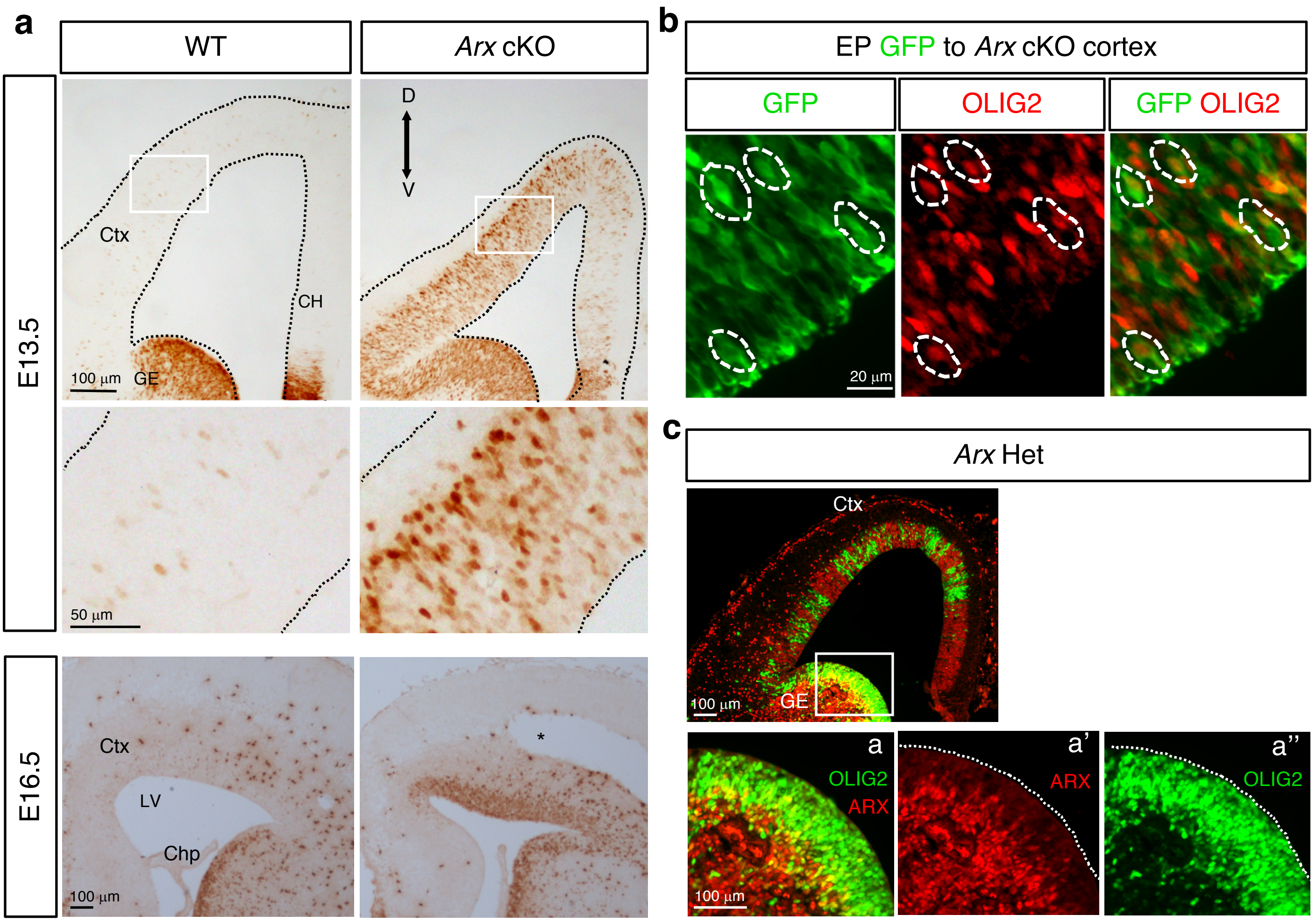

Supplementary Fig S1. a, Representative images of embryonic neocortex of the *Arx*$^{+/y}$ (WT) and *Arx*$^{cKO/y}$ (*Arx* cKO) mice at E13.5 and E16.5 (coronal sections) immunolabeled with OLIG2 antibody. * indicates tissue damage artifact. D-V indicates dorsal-ventral axis. The middle panels are magnified images of the boxed areas in the top panels. **b,** Representative images of the VZ of the *Arx* $^{cKO/y}$ embryonic neocortex electroporated with GFP expression construct (*pCIG*) (EP at E13.5 and harvested at E14.5) and double immunolabeled with OLIG2 and GFP, suggest that OLIG2+ cells in cKO are originated dorsally not migrated from the ventral telencephalon. Dotted circles mark examples of OLIG2 and GFP double positive cells. **c,** Representative images of embryonic forebrain (E14.5) of the *Arx*$^{cKO/+}$ female (Arx Het) double immunolabeled with OLIG2 and ARX antibodies (a-a'' are magnified images of the boxed area in the upper panel). Dotted lines mark the ventricular surface. Note that the upper panel image is the same image used in Fig. 2a. CH, cortical hem; Chp, choroid plexus primordium; Ctx, neocortex; GE, ganglionic eminence; LV, lateral ventricle

Changes in ventral and dorsal genes in the *Arx* $^{-/y}$ cortex; but no changes in *Shh* level in *Arx* cKO

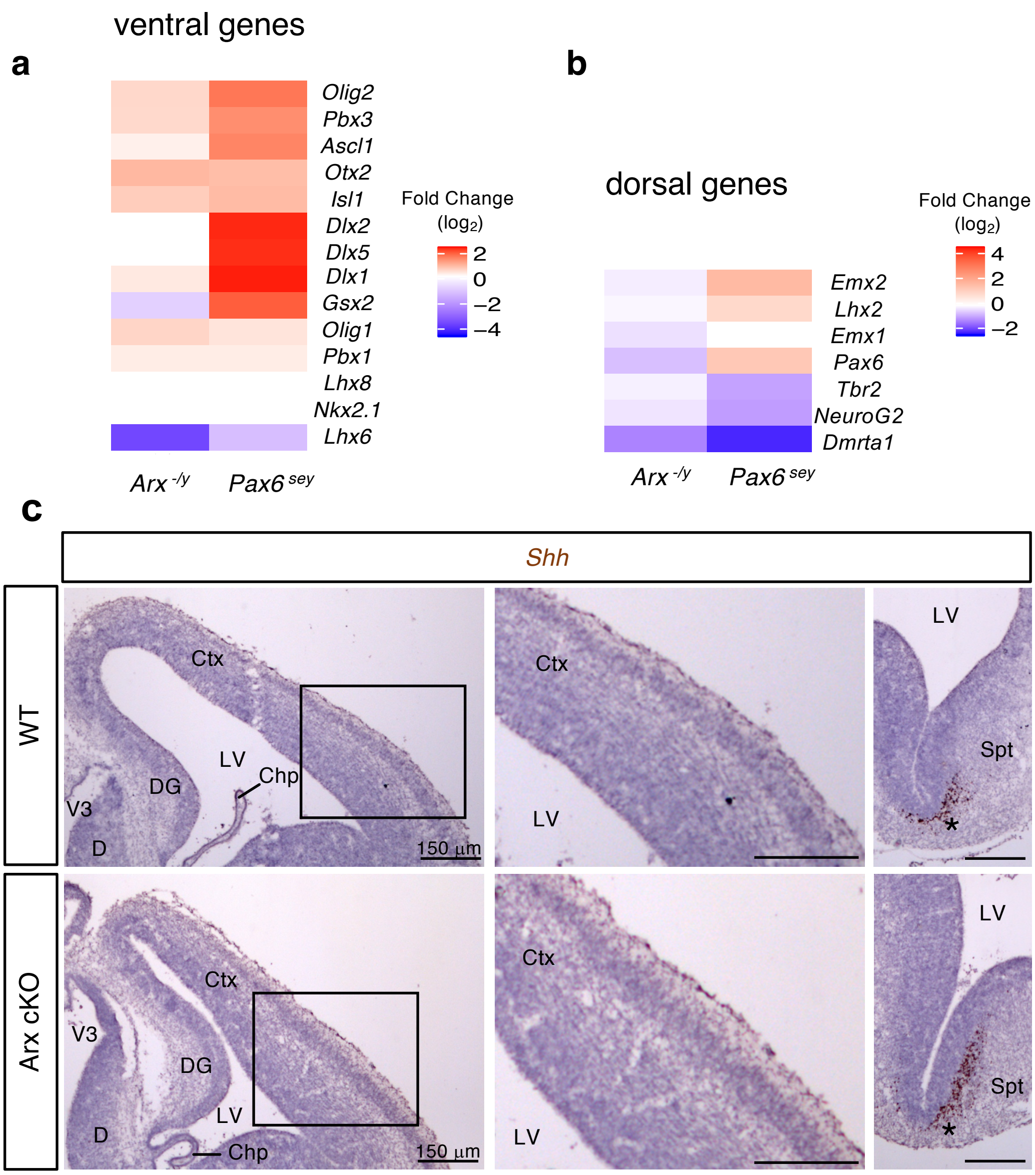

Supplementary Fig S2. a-b, Heatmap of microarray data showing upregulated ventral genes (a) and downregulated dorsal genes (b) in the cortex of *Arx* (*Arx* $^{-/y}$) and *Pax6* (*Pax6* ^{sey}) deficient mice (E14.5), using published data[1, 2]. A similar upregulation of ventral genes was identified in *Arx* mutant mice as well as in *Pax6* mutant mice that also show defective cortical development with overlapping features to those observed in the *Arx* $^{-/y}$ cortex[2, 3]. **c,** Representative images of *Shh* RNA *in situ* hybridization in WT or *Arx* cKO brain sections (coronal) (E14.5). In the cortex (left and middle panels), no transcripts were detected in either genotype, while the ventral expression (* in right panels) appeared the same in both genotypes. Middle panels are magnified images of the boxed areas in left panels. Note that the difference in *Shh* transcript levels in WT and cKO ventral forebrains is due to slightly different section levels. Chp, choroid plexus primordium; Ctx, neocortex; D, dorsal thalamus; DG, dentate gyrus; LV, lateral ventricle; Spt, septum; V3, third ventricle.

Upregulation of SHH targets, *Gli1* and *Ptch1*, in Arx cKO

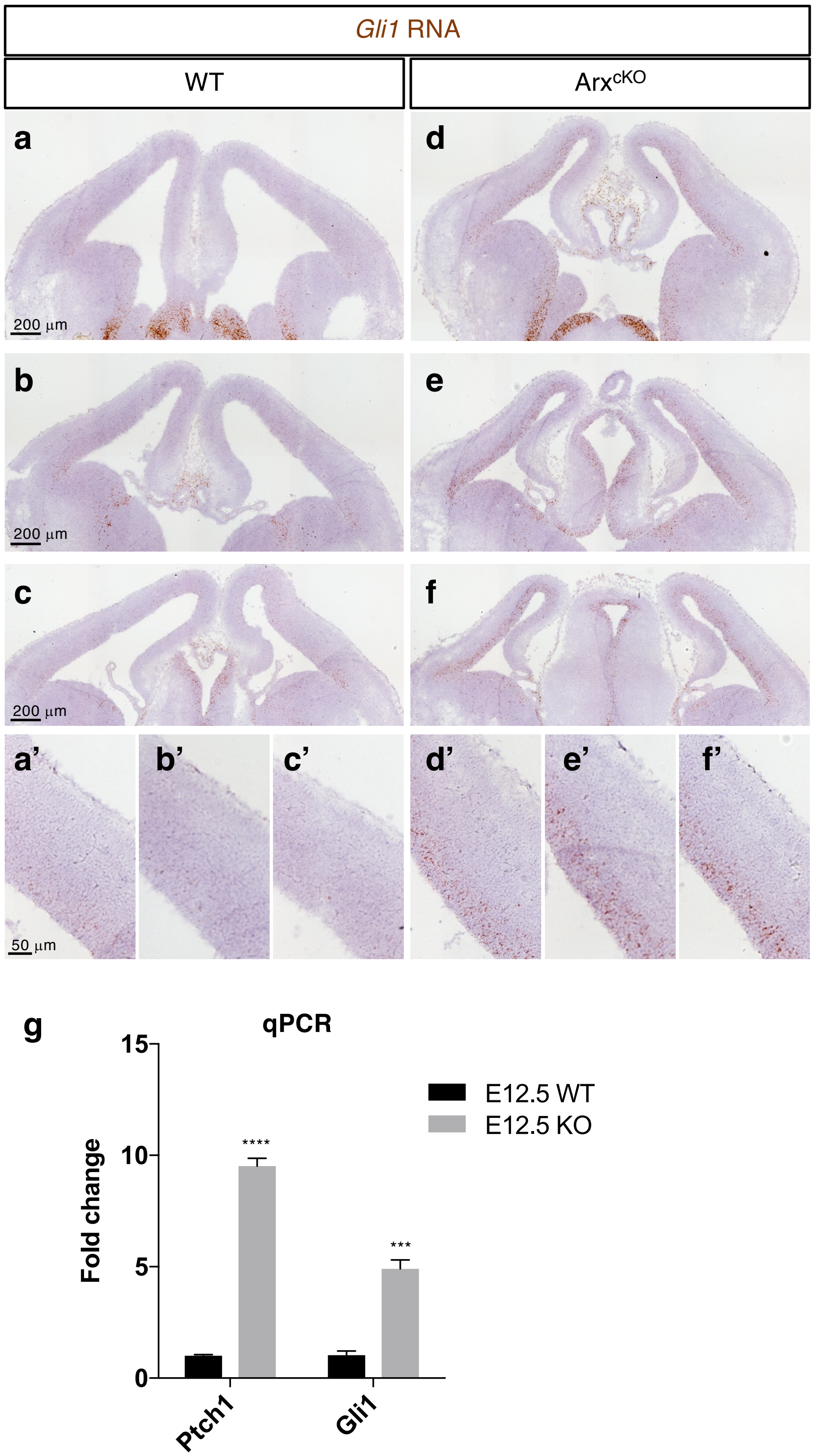

Supplementary Fig S3. a-f, Serial sections of WT (**a, c, e**) and *Arx* cKO (**b, d, f**) embryonic brains with *Gli1* RNA *in situ* hybridization. **a'-f',** Magnified images of the boxed areas in **a-f**. **g,** RT-qPCR for SHH targets, *Ptch1* and *Gli1,* using E12.5 WT and $Arx^{cKO/y}$ cortices. Error bars: mean ± s.e.m (n=6 per sample; ****, P<0.0001; ***, P=0.002; unpaired t-test).

Olig2 overexpression does not change the level of *Cdkn1c* (p57/Kip2)

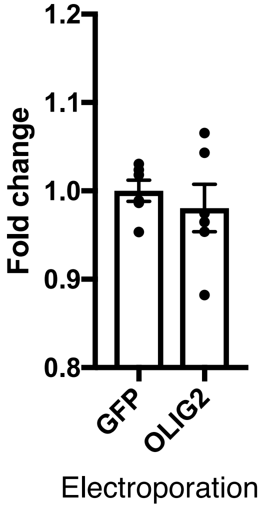

Supplementary Fig S4. RT-qPCR results for *Cdkn1c* (also known as p57/Kip2) in *Olig2* (pCIG-*Olig2*) vs GFP (pCIG) electroporated brains (EP at E13.5 and harvested at E14.5). No significant difference was detected. Error bars: mean ± s.e.m (n=6 from two EPed brains per sample; unpaired t-test).

Supplementary References

1. Colasante, G. *et al.* ARX regulates cortical intermediate progenitor cell expansion and upper layer neuron formation through repression of Cdkn1c. *Cerebral Cortex* **25,** 322–335 (2015).

2. Walcher, T., Xie, Q., Sun, J., Irmler, M., Beckers, J., Ozturk, T., et al. Functional dissection of the paired domain of Pax6 reveals molecular mechanisms of coordinating neurogenesis and proliferation. *Development*, *140*(5), 1123–1136 (2013).

3. Manuel, M. N., Mi, D., Mason, J. O. & Price, D. J. Regulation of cerebral cortical neurogenesis by the Pax6 transcription factor. *Front. Cell. Neurosci.* **9,** 28 (2015).
